# Intrauterine phenotype, genetic analysis, and pregnancy follow-up of fetuses with the 16p12.2 microdeletion

**DOI:** 10.3389/fgene.2025.1595399

**Published:** 2025-09-24

**Authors:** Meiying Cai, Na Lin, Hailong Huang, Wenqiang You, Nan Guo, Liangpu Xu

**Affiliations:** ^1^ Medical Genetic Diagnosis and Therapy Center, Fujian Maternity and Child Health Hospital College of Clinical Medicine for Obstetrics & Gynecology and Pediatrics, Fujian Key Laboratory for Prenatal Diagnosis and Birth Defect, Fujian Clinical Research Center for Maternal-Fetal Medicine, National Key Obstetric Clinical Specialty Construction Institution of China, Fujian Medical University, Fuzhou, China; ^2^ Fujian Maternity and Child Health Hospital, College of Clinical Medicine for Obstetrics & Gynecology and Pediatrics, Fujian Medical University, Fuzhou, China

**Keywords:** 16p12.2 microdeletion, intrauterine phenotype, fetus, SNP array, follow-up

## Abstract

Reports on the intrauterine phenotype of the 16p12.2 microdeletion are few. A retrospective analysis of the clinical data, genetic testing results, and neonatal prognoses of fetuses with the 16p12.2 microdeletion was conducted to provide a basis for their clinical management. The research participants were pregnant women who underwent prenatal diagnoses between November 2016 and June 2024. Among them, 12,000 cases were selected for karyotype analyses and single-nucleotide polymorphism (SNP) array testing. In the SNP array, 13 out of 12,000 fetuses (0.1%) had the 16p12.2 microdeletion, which included 6 cases of distal deletions and 7 of proximal deletions, involving fragment sizes ranging from 511 to 994 kb. The 16p12.2 distal deletion mainly involves the *OTOA* gene, whereas the 16p12.2 proximal deletion mainly involves the *EEF2K* and *CDR2* genes. Among the 13 fetuses, five exhibited intrauterine phenotypes, including a small biparietal diameter, head circumference cerebellar dysplasia, corpus callosum dysplasia, small abdominal circumference, mild ventriculomegaly, left ventricular hyperechoic foci, small kidney measurements, nasal bone dysplasia, and polyhydramnios. The inheritance testing of six cases revealed that one case was *de novo* and five were inherited from the father/mother with normal phenotypes. Except for one case of early abortion, two cases of fetal ultrasound abnormality-led terminations, and one of adverse pregnancy history-based termination, the remaining nine cases included full-term delivery and no significant abnormalities in the birth conditions. One case was lost at follow-up during a phone call 6 months after birth, and the remaining eight infants did not show any significant abnormalities during follow-up. The SNP array effectively diagnosed the 16p12.2 microdeletion, recognized its range and associated genes, and improved the prenatal diagnoses. Thirteen 16p12.2 microdeletion-carrying fetuses lacked intrauterine-specific phenotypes, and eight showed no abnormalities during the most recent postnatal follow-up. However, considering delays in the children’s hearing and neurological development, it is important to conduct continuous and regular post-birth follow-ups. When 16p12.2 deletions are inherited or restricted to distal regions, they often exhibit reduced penetrance. This underscores the need for cautious interpretations of prenatal genetic data.

## 1 Introduction

Human chromosome 16 is a small centromeric chromosome belonging to Group E, with a DNA length of 90.4 Mb. Ten percent of its genome is composed of repetitive sequences that can be easily rearranged through the recurrence mechanism of non-allelic homologous recombination, leading to genomic instability (Redaelli et al., 2020). The 16p12.2 microdeletion can be categorized into distal and proximal deletions. The 16p12.2 distal deletion is defined as the position ranging from 21,570,113 to 21,740,423 in the reference genome (NCBI Build GRCh37/hg19) and is associated with autosomal recessive non-syndromic hearing loss (Shahin et al., 2010). The 16p12.2 proximal deletion is defined as the position ranging from 21,948,445 to 22,430,805 in the reference genome (NCBI Build GRCh37/hg19). The recurrent deletion of 16p12.2 occurs in this region with a 520-kb deletion, and it is characteristic of clinical manifestations that are variable and do not constitute a recognizable syndrome (Uppinkudru et al., 2024). Patients with the 16p12.2 proximal deletion often exhibit developmental delays, varying degrees of cognitive impairment, short stature, heart malformation, epilepsy, and mental and/or behavioral abnormalities. Other possible issues include hearing loss, dental abnormalities, kidney abnormalities, male genital abnormalities, and cleft lips or palates (Uppinkudru et al., 2024; Pagon et al., 1993). The phenotype of individuals carrying larger or smaller deletions in this region may differ clinically from that of individuals with the 16p12.2 deletion.

The 16p12.2 microdeletion has not been systematically described in prenatal cases owing to the limitations of phenotype identification in prenatal diagnoses. Fortunately, this microdeletion has been increasingly observed during chromosome microarray analyses for prenatal testing (Karen and Brynn, 2016; Xiang et al., 2020). Because of its incomplete penetrance and variable expression, the genetic counseling of the potential phenotype caused by this deficiency is challenging. Single-nucleotide polymorphism (SNP) arrays can be used to detect genome-wide copy number variations (CNVs) and the loss of heterozygosity (Brady and Vermeesch, 2012; Kamath et al., 2022). The present study reports the prenatal diagnoses of 13 fetuses with the 16p12.2 microdeletion and retrospectively analyzes their prenatal diagnostic indications, prenatal ultrasound findings, chromosome karyotypes, genetics, variation tracing, pregnancy outcomes, and post-birth follow-ups to create a basis for prenatal diagnoses and genetic counseling.

## 2 Participants and methods

### 2.1 Participants

This was a retrospective study. The research participants were pregnant women who underwent prenatal diagnoses at Fujian Maternal and Child Health Hospital between November 2016 and June 2024. In total, there were 12,000 cases of patients who underwent both karyotype and SNP array analyses. The average age of the pregnant women was 28.4 years old (range: 17–46 years old), and the average gestational age was 24.2 weeks (range: 12–38 weeks). All the pregnant women received genetic counseling and signed informed consent forms before undergoing invasive diagnoses. This study was approved by the Ethics Committee of Fujian Maternal and Child Health Hospital (approval no. 2021KRD09001), and all the parents who allowed examinations of fetal data signed informed consent forms.

### 2.2 Chromosome karyotype analysis

Ultrasound was used to perform chorionic villus sampling through abdominal puncture, amniocentesis, or umbilical cord blood puncture based on the gestational ages of the participants. Chorionic villus sampling was performed at 11–13+6 weeks of pregnancy, amniocentesis at 18–24+6 weeks, and umbilical cord blood puncture after 25 weeks. Cell culture, mid-term chromosome division phase preparation, and G-banding karyotype analyses were performed according to conventional methods. Thirty karyotypes were counted for each case, and if chromosomal mosaicism was detected, the count was increased to 50. In chorionic villus sampling, the 30 metaphases were counted from short-term cultures (direct preparations), as these provide rapid results and are standard for initial cytogenetic analyses of chorionic villi. In the amniotic fluid analyses, the 30 metaphases were counted from flask cultures.

### 2.3 Single-nucleotide polymorphism array

The experimental procedures were strictly performed in accordance with the standard procedures of the SNP array analysis, which was performed using the Affymetrix CytoScan™ 750K Array platform (manufacturer: Affymetrix, Thermo Fisher Scientific, United States), which provides genome-wide coverage with ∼750,000 markers (including SNPs and copy number probes) at a median resolution of ∼10 kb. Sample DNA was hybridized to the array following the manufacturer’s standard protocols. For CNV detection and calling, we applied the following criteria: log2 ratio thresholds: gains (>0.25) and losses (<−0.25) with ≥50 consecutive probes. Chromosome Analysis Suite (ChAS) software v4.2 (Affymetrix) was used for primary analyses, including quality control, SNP/CNV visualization, and scatter plot-based copy number profiling. The bioinformatics filtering included the following: CNVs were cross-referenced against public databases, including DGV, DECIPHER, OMIM, and the UCSC Genome Browser, and annotated using the ACMG/ClinGen guidelines. CNVs were classified as pathogenic, likely pathogenic, benign, likely benign, or VUS based on the ACMG standards (Riggs et al., 2020), integrating evidence from population frequency, gene content, and functional impact. The sample QC thresholds included the following: sample call rate: ≥97% (samples with lower call rates were excluded); gender concordance: verified by X chromosome heterozygosity and marker consistency; contamination check: samples with ≥5% contamination (inferred from B-allele frequency noise) were discarded. The CNV interpretation criteria included the following: size threshold: CNVs <50 kb were excluded unless they were overlapping clinically relevant genes.

### 2.4 Genetic counseling for the SNP array results

Owing to differences in the penetrance and expression of many detected genetic diseases, significant variations in clinical manifestations may occur among different patients (D’Alessandro et al., 2014). When an SNP array indicates a pathogenic CNV, professional genetic counseling is a requisite. If the pathogenic CNV is a clear chromosomal microdeletion or microduplication syndrome and the parents have plans for another pregnancy, they are recommended to undergo SNP array testing to determine whether the CNV is *de novo* and to assess the risk for future pregnancies.

### 2.5 Pregnancy outcomes and postnatal follow-up

Clinical data were obtained from the prenatal diagnoses and follow-up records. Pregnancy outcomes and individual growth and development were tracked through telephone follow-ups, which were conducted until December 2024.

## 3 Results

### 3.1 Karyotype analysis

The karyotype test results of the 13 fetuses were all normal.

### 3.2 Single-nucleotide polymorphism array

**FIGURE 1:**
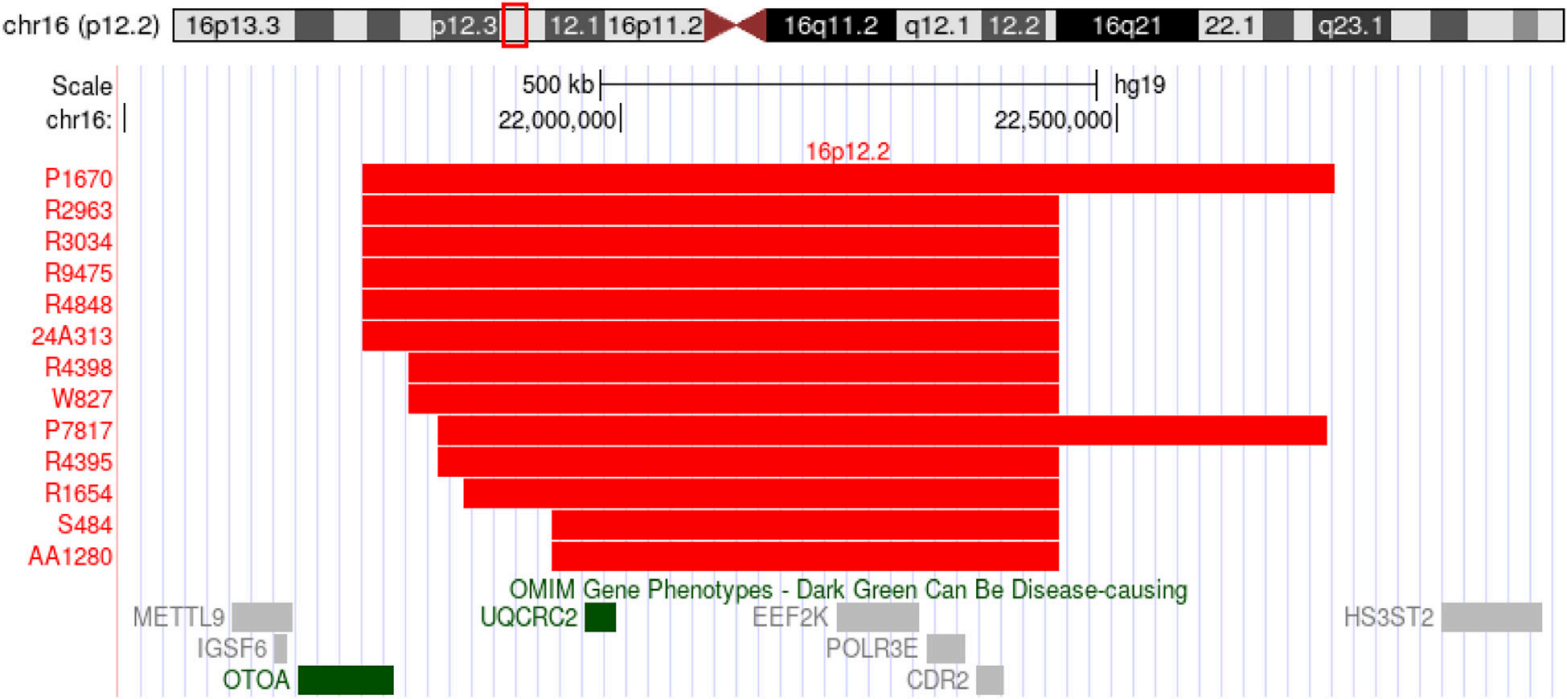
SNP-array shows 13 fetuses with deletions in the 16p12.2 region.

In the SNP array, 13 out of 12,000 fetuses (0.1%) had a 16p12.2 microdeletion, which included 6 cases of distal deletions and 7 of proximal deletions, involving fragment sizes ranging from 511 to 994 kb. The 16p12.2 distal deletion mainly involves the OTOA gene, whereas the 16p12.2 proximal deletion mainly involves the EEF2K and CDR2 genes (Figure 1).

### 3.3 Prenatal diagnostic indications and parental tracing of fetuses with the 16p12.2 microdeletion

Among the 13 cases with the prenatal diagnostic indicators in fetuses, three were of advanced age, two had adverse pregnancy history, two involved high-risk serological screening, one showed non-invasive prenatal testing (NIPT) positivity, and the remaining five had prenatal ultrasound abnormalities. Among the five cases with prenatal ultrasound abnormalities, one case had a biparietal diameter less than the normal predicted value of M-1.6 SD and head circumference less than the normal predicted value of M-1.9 SD; one had a biparietal diameter and head circumference less than M-2SD and cerebellar and corpus callosum dysplasia; one exhibited abdominal circumference at the normal predicted value of M-3.4 SD; one had mild ventriculomegaly, left ventricular hyperechoic lesion, and the bilateral kidney measurements were smaller; and one case had nasal bone dysplasia and polyhydramnios (Table 1).

**TABLE 1:** Clinical characteristics and follow-up of fetuses with 16p12.2 microdeletion.

Case	Prenatal diagnosis pointer	SNP arrary	Cassification	Size (Kb)	Contains genes	Hereditary	Pregnancy outcome	Follow-up
P1670	NIPT positivity	arr[hg19]16p12.2(21,740,199-22,718,351)x1	Distal deletion	978	OTOA, UQCRC2, EEF2K, POLR3E, CDR2	Refuse	Eutocia, healthy	Loss to follow-up
R2963	Advanced age	arr[hg19]16p12.2(21,740,199-22,442,007)x1	Distal deletion	702	OTOA, UQCRC2, EEF2K, POLR3E, CDR2	Refuse	Eutocia, healthy	3 years, healthy
R3034	Advanced age	arr[hg19]16p12.2(21,740,199-22,442,007)x1	Distal deletion	702	OTOA, UQCRC2, EEF2K, POLR3E, CDR2	Mat	Eutocia, healthy	3 years, healthy
R9475	Adverse pregnancy history	arr[hg19]16p12.2(21,740,200-22,442,007)x1	Distal deletion	702	OTOA, UQCRC2, EEF2K, POLR3E, CDR2	Refuse	TP	-
R4848	Biparietal diameter and head circumference less than M-2SD,cerebellar dysplasia, corpus callosum dysplasia	arr[hg19]16p12.2(21,740,200-22,442,007)x1	Distal deletion	702	OTOA, UQCRC2, EEF2K, POLR3E, CDR2	*Denovo*	TP	-
24A313	High-risk serological screening	arr[hg19]16p12.2(21,740,200-22,442,007)x1	Distal deletion	702	OTOA, UQCRC2, EEF2K, POLR3E, CDR2	Pat	Eutocia, healthy	6 months, healthy
AA1280	Advanced age	arr[hg19]16p12.2(21,931,248-22,442,007)x1	Proximal deletion	511	UQCRC2, EEF2K, POLR3E, CDR2	Refuse	Eutocia, healthy	10 months, healthy
R1654	High-risk serological screening	arr[hg19]16p12.2(21,841,353-22,442,007)x1	Proximal deletion	601	UQCRC2, EEF2K, POLR3E, CDR2	Refuse	Eutocia, healthy	4 years 6 months, healthy
W827	Adverse pregnancy history	arr[hg19]16p12.2(21,787,031-22,442,007)x1	Proximal deletion	655	UQCRC2, EEF2K, POLR3E, CDR2	Pat	Early abortion	-
R4398	Biparietal diameter less than the normal predicted value of M-1.6 SD, head circumference less than the normal predicted value of M-1.9 SD	arr[hg19]16p12.2(21,787,031-22,442,007)x1	Proximal deletion	655	UQCRC2, EEF2K, POLR3E, CDR2	Mat	TP	-
S484	Abdominal circumference at the normal predicted value of M-3.4 SD	arr[hg19]16p12.2(21,931,248-22,442,007)x1	Proximal deletion	511	UQCRC2, EEF2K, POLR3E, CDR2	Refuse	Eutocia, healthy	1 year 6 months, healthy
R4395	Mild ventriculomegaly, left ventricular hyperechoic lesion, bilateral kidney measurements were smaller	arr[hg19]16p12.2(21,816,543-22,441,367)x1	Proximal deletion	625	UQCRC2, EEF2K, POLR3E, CDR2	Refuse	Eutocia, healthy	2 years 8 months, healthy
P7817	Nasal bone dysplasia and polyhydramnios	arr[hg19]16p12.2(21,816,542-22,710,614)x1	Proximal deletion	994	UQCRC2, EEF2K, POLR3E, CDR2	Pat	Eutocia, healthy	5 years 8 months, healthy

NIPT, non invasive prenatal testing; TP, termination of pregnancy.

After professional genetic counseling, the parents of the six fetuses with the 16p12.2 microdeletion agreed to undergo pedigree analyses. One fetus had a *de novo* 16p12.2 microdeletion, two inherited from fathers with normal phenotypes, and three were derived from mothers with normal phenotypes. The parents of the remaining seven 16p12.2 microdeletion-carrying fetuses refused inheritance testing (Table 1).

### 3.4 Pregnancy outcomes and follow-up of fetuses with the 16p12.2 microdeletion

Except for one case of early abortion, two of fetal ultrasound abnormalities (one case with a small biparietal diameter and head circumference and the other with cerebellar and corpus callosum dysplasia in addition to a small biparietal diameter and head circumference), and one of adverse pregnancy history, all the pregnancies were terminated. The remaining nine patients were pregnant and underwent full-term delivery, and no significant abnormalities were observed in the birth conditions. One case was lost at follow-up during a phone call 6 months after birth, and the remaining eight infants did not show any significant abnormalities during follow-up (Table 1).

## 4 Discussion

Chromosomal microdeletions and microduplications are chromosomal diseases with complex clinical manifestations caused by the loss or duplication of small fragments of chromosomes (generally <10Mb, accounting for 0.01%–0.02% of the entire genome; hence, they cannot be detected by karyotype analyses), which result in changes in normal gene dosage (Capalbo et al., 2017). Common clinical manifestations of microdeletions and duplications include intellectual disability, abnormal growth and development, distinctive facial features, visceral organ deformities, endocrine disorders, changes in mental and behavioral states, and tumors (Park et al., 2019). At present, nearly 300 types of such diseases have been identified, with an incidence rate ranging from 1/200,000 to 1/4,000 and a combined incidence rate of nearly 1/600 (Anja et al., 2012). The incidence of pathogenic or potentially pathogenic chromosomal microdeletions and duplications is 1.7%, and the risk of recurrence is high. Most chromosomal microdeletion and duplication diseases involve new mutations, accounting for 85%–95% of cases, with familial inheritance accounting for 5%–10% of cases, and the risk of onset is not significantly correlated with age (Nevado et al., 2014; Coe et al., 2014). The number of genes covered by microdeletions and duplications may not be very large, resulting in diseases that are slightly less effective than whole-chromosome diseases. Generally, such aberrations do not lead to miscarriages and may cause abnormal organ development during fetal development, leading to birth defects; however, more serious cases of microdeletion syndrome may lead to intellectual and intellectual disability, and the children may be unable to take care of themselves and require lifelong care (Goldenberg, 2018; Watson et al., 2014).

With the continuous development of molecular diagnostic technology, novel microdeletions and microduplications are being discovered, including the 16p12.2 microdeletion. The 16p12.2 microdeletion involves both distal and proximal deletions. Proximal deletions are associated with neurodevelopmental disorders, particularly intellectual disability and developmental delay (Shahin et al., 2010). Distal deletions (extending beyond ∼22.5 Mb into 16p12.1) demonstrate variable expression and reduced penetrance, with some carriers showing minimal phenotypic consequences (Pagon et al., 1993). In this study, 13 out of 12,000 fetuses (0.1%) had a 16p12.2 microdeletion, which included 6 cases of distal deletions and 7 of proximal deletions. The 16p12.2 distal deletion mainly involves the OTOA gene, which is a recessive non-syndromic deafness-causing gene on the autosomes (Sugiyama et al., 2019). The 16p12.2 proximal deletion mainly involves the UQCRC2, EEF2K, POLR3E, and CDR2 genes. EEF2K is associated with learning and memory, synaptic plasticity, and the short-term antidepressant effects of ketamine (Mccamphill et al., 2015). CDR2 is highly expressed in cerebellar Purkinje cells. Its absence in animal models has been reported to result in motor impairments (Peterson et al., 1992). POLR3E is a core component of RNA polymerase III, responsible for transcribing non-coding RNAs, such as tRNA, and is crucial for cell proliferation and development. Mutations in POLR3E are associated with hypomyelination cerebral white matter disorder. UQCRC2 encodes the core protein of mitochondrial complex III, which affects oxidative phosphorylation and ATP production. Mutations in UQCRC2 are associated with mitochondrial complex III deficiency. The 16p12.2 microdeletion is a neurodevelopmental susceptibility site with a reported penetrance of approximately 12.3% (Rosenfeld et al., 2013). Its clinical phenotypes can vary and manifest as developmental delay, mild-to-moderate intellectual disability, language delay, mental and behavioral abnormalities, microcephaly, congenital heart defects, sleep disorders, epilepsy, and other abnormalities (D’Alessandro et al., 2014; Wang et al., 2022). The 16p12.2 microdeletion is inherited in an autosomal dominant manner, with up to 95% of deletions inherited from the parents. If one of the parents is heterozygous with a 16p12.2 microdeletion, the risk of genetic deletion in the siblings of the proband in the family is the same as that of autosomal dominant inheritance (i.e., 50%); however, due to incomplete clinical expression, the risk of clinical expression in siblings should be less than 50%. Children with a family history of impaired neurological development or mental illness are likely to present severe clinical phenotypes. Missing fragments have also been reported in normal randomized controls without phenotypic features and unaffected relatives (Butler, 2020). Few cases of the 16p12.2 microdeletion in prenatal diagnoses have been reported. There are reports that the intrauterine ultrasound phenotype of fetuses with the 16p12.2 microdeletion is characterized by marked growth retardation and cardiomyopathy (Stabile et al., 2023; Leung et al., 2021). Among the 13 16p12.2 microdeletion-carrying fetuses in this study, five cases showed ultrasound abnormalities, including two with a small head circumference. After tracing the family lineage, one case was found to be inherited from a mother with a normal phenotype and the other was *de novo*. After fully informing the participant of the risks, the participant and her family chose to terminate the pregnancy. In one case, the ultrasound showed nasal bone dysplasia and polyhydramnios in the fetus, and inheritance testing showed that it was inherited from a father with a normal phenotype. The ultrasound detected one fetus with a small abdominal circumference and one fetus with a widened right ventricle, strong echogenicity in the left ventricle, and small kidney measurements. The pregnant women and their families refused to trace the origin of the pregnancy. After fully informing them of the risks, they chose to continue the pregnancy until full-term delivery. These two infants were followed up with at birth and again 7 months after birth, and no significant abnormalities were observed in their development. Among the eight fetuses with no abnormalities detected by ultrasound, one case involved an early abortion, one was terminated, and the remaining six underwent full-term delivery. At birth and 8 months after birth, except for one case that was lost at follow-up, no significant abnormalities were found in the development of the other five cases. However, due to differences in the degree of expression of the 16p12.2 microdeletion, attention must be paid to the development of the nervous system during individual growth and development.

Among the 13 fetuses in this study, six with the 16p12.2 microdeletion carried UQCRC2. In addition to the deletion of the EEF2K, POLR3E, and CDR2 genes, there was a deletion of the OTOA gene. OTOA is associated with an autosomal recessive non-syndromic hearing loss phenotype (Sugiyama et al., 2019). OTOA encodes otoancorin, which is essential for inner ear mesentery (Kim et al., 2019). The variation in OTOA gene expression is associated with autosomal recessive deafness-22 (DFNB22) (Shahin et al., 2010; Tassano et al., 2019; Swetha et al., 2025). DFNB22 is a form of non-syndromic sensorineural hearing loss caused by damage to the inner ear nerve receptors, neural pathways leading to the brain, or brain regions that receive sound information. Among the six fetuses with the OTOA gene deletion, one had an intrauterine ultrasound phenotype of biparietal diameter, small head circumference, cerebellar dysplasia, and corpus callosum dysplasia. Through inheritance testing, the mutation was determined to be *de novo*. After fully informing the participant of the risks, the participant and her family chose to terminate the pregnancy. Two patients had normal intrauterine ultrasound phenotypes; however, the participants and their families refused to record their family histories. After fully informing them of the risks, the participants and their families chose to terminate the pregnancy. There were three remaining fetuses, of which two had been traced by their families and found to inherit the 16p12.2 deletion from mothers/fathers with normal phenotypes, and one whose family refused to undergo tracing but were fully informed of the risks and chose to continue pregnancy until full-term delivery. The data showed that inherited 16p12.2 deletions were more likely to have mild/no phenotypes compared to those in *de novo* cases. The distal 16p12.2 region has been linked to lower penetrance. No symptoms were observed at or after birth. In cases with an OTOA gene deletion, it was recommended that parents monitor the hearing status of their offspring after birth. In our cohort, OTOA deletions were identified in six fetuses. While biallelic OTOA loss has been linked to DFNB22 deafness, the heterozygous deletions observed herein may contribute to variable auditory phenotypes, particularly if combined with additional genetic or environmental factors. Prenatal assessments of auditory function remain challenging, but postnatal follow-up in these cases may clarify the penetrance of OTOA-related hearing loss.

This study had several limitations. The number of cases was relatively small, as only a small portion of the population was surveyed. The short follow-up period may further have been followed by undetected intellectual or learning disabilities and behavioral and hearing problems. Therefore, the follow-up period in future studies should be extended to enable a more comprehensive assessment of growth and development.

## 5 Conclusion

SNP array testing can effectively detect the 16p12.2 microdeletion, clarify its deletion range and the genes it contains, and improve prenatal diagnoses. In this study, 13 fetuses with the 16p12.2 microdeletion lacked intrauterine-specific phenotypes, and three fetuses carrying normal inherited phenotypes were carried to full-term. Follow-ups soon after birth did not reveal abnormalities; therefore, family comparisons can help reduce unnecessary active terminations of pregnancy. Given that a child’s hearing and neurological development may be delayed, it is important to conduct continuous and regular follow-ups after birth. Currently, only a few studies on the prenatal 16p12.2 microdeletion can be retrieved, and there is a lack of specific phenotypes in infants before birth. We will continue to accumulate relevant data to provide a better basis for genetic counseling.

## Data Availability

The original contributions presented in the study are included in the article/supplementary material, further inquiries can be directed to the corresponding authors.

## Abbreviations

SNP, single-nucleotide polymorphism; CNV, copy number variation; NIPT, non-invasive prenatal testing; DFNB22, autosomal recessive deafness-22.

## References

[B1] Anja W. Mrasek K. Klein E. Mulatinho M. Llerena J. C. Jr Hardekopf D. (2012). Microdeletion and microduplication syndromes. J. Histochem Cytochem 60 (5), 346–358. 10.1369/0022155412440001 22396478 PMC3351230

[B2] Brady P. D. Vermeesch J. R. (2012). Genomic microarrays: a technology overview. Prenat. Diag 32, 336–343. 10.1002/pd.2933 22467164

[B3] Butler M. G. (2020). Imprinting disorders in humans: a review. Curr. Opin. Pediatr. 32, 719–729. 10.1097/MOP.0000000000000965 33148967 PMC8791075

[B4] Capalbo A. Rienzi L. Ubaldi F. M. (2017). Diagnosis and clinical management of duplications and deletions. Fertil. Steril. 107, 12–18. 10.1016/j.fertnstert.2016.11.002 28040093

[B5] Coe B. Witherspoon K. Rosenfeld J. van Bon B. W. M. Vulto-van Silfhout A. T. Bosco P. (2014). Eichler EE: refining analyses of copy number variation identifies specific genes associated with developmental delay. Nat. Genet. 46, 1063–1071. 10.1038/ng.3092 25217958 PMC4177294

[B6] D’Alessandro L. C. A. Werner P. Xie H. M. Hakonarson H. White P. S. Goldmuntz E. (2014). The prevalence of 16p12.1 microdeletion in patients with left-sided cardiac lesions. Congenit. Heart Dis. 9, 83–86. 10.1111/chd.12097 23682798 PMC4575124

[B7] Goldenberg P. (2018). An update on common chromosome microdeletion and microduplication syndromes. Pediatr. Ann. 47, e198–e203. 10.3928/19382359-20180419-01 29750287

[B8] Kamath V. Chacko M. P. Kirubakaran R. Mascarenhas M. Kamath M. S. (2022). Single nucleotide polymorphism array versus karyotype for prenatal diagnosis in fetuses with abnormal ultrasound: a systematic review and meta-analysis. Eur. J. Obstet. 271, 235–244. 10.1016/j.ejogrb.2022.02.011 35245714

[B9] Karen W. Brynn W. R. J. (2016). Chromosomal microarrays for the prenatal detection of microdeletions and microduplications. Clin. Lab. Med. 36, 261–276. 10.1016/j.cll.2016.01.017 27235911

[B10] Kim B. J. Kim Dy Han J. H. Oh J. Kim A. R. Lee C. (2019). Clarification of glycosylphosphatidylinositol anchorage of OTOANCORIN and human OTOA variants associated with deafness. Hum. Mutat. 40, 525–531. 10.1002/humu.23719 30740825 PMC6467692

[B11] Leung C. Engineer A. Kim M. Y. Lu X. Feng Q. (2021). Myocardium-specific deletion of Rac1 causes ventricular noncompaction and outflow tract defects. J. Cardiovasc Dev. Dis. 8, 29. 10.3390/jcdd8030029 33804107 PMC8001666

[B12] Mccamphill P. K. Farah C. A. Anadolu M. N. Hoque S. Sossin W. S. (2015). Bidirectional regulation of eEF2 phosphorylation controls synaptic plasticity by decoding neuronal activity patterns. J. Neurosci. 35, 4403–4417. 10.1523/JNEUROSCI.2376-14.2015 25762683 PMC6605292

[B13] Nevado J. Mergener R. Palomares-Bralo M. Souza K. R. Vallespín E. Mena R. (2014). New microdeletion and microduplication syndromes: a comprehensive review. Genet. Mol. Biol. 37, 210–219. 10.1590/s1415-47572014000200007 24764755 PMC3983590

[B14] Pagon R. A. Adam M. P. Ardinger H. H. (1993). 16p12.2 recurrent deletion. In: GeneReviews®. Seattle, WA: University of Washington. 25719193

[B15] Park H. Chun S. M. Shim J. Oh J. H. Cho E. J. Hwang H. S. (2019). Detection of chromosome structural variation by targeted next-generation sequencing and a deep learning application. Sci. Rep. 9, 3644. 10.1038/s41598-019-40364-5 30842562 PMC6403216

[B16] Peterson R. Rosenblum M. K. Kotanides H. Posner J. B. (1992). Kotanides and Posner: paraneoplastic cerebellar degeneration. I. A clinical analysis of 55 anti-Yo antibody-positive patients. Neurology 42, 1931–1937. 10.1212/wnl.42.10.1931 1407575

[B17] Redaelli S. Maitz S. Crosti F. Sala E. Villa N. Spaccini L. (2020). Refining the phenotype of recurrent rearrangements of chromosome 16. Int. J. Mol. Sci. 20, 1095–10. 10.3390/ijms20051095 30836598 PMC6429492

[B18] Riggs E. R. Andersen E. F. Cherry A. M. Kantarci S. Kearney H. Patel A. (2020). Technical standards for the interpretation and reporting of constitutional copy-number variants: a joint consensus recommendation of the American College of Medical Genetics and Genomics (ACMG) and the Clinical Genome Resource (ClinGen). Genet. Med. 22, 245–257. 10.1038/s41436-019-0686-8 31690835 PMC7313390

[B19] Rosenfeld J. A. Coe B. P. Eichler E. E. Cuckle H. Shaffer L. G. (2013). Estimates of penetrance for recurrent pathogenic copy-number variations. Genet. Med. 15, 478–481. 10.1038/gim.2012.164 23258348 PMC3664238

[B20] Shahin H. Walsh T. Rayyan A. A. Lee M. K. Higgins J. Dickel D. (2010). Five novel loci for inherited hearing loss mapped by SNP-based homozygosity profiles in Palestinian families. Eur. J. Hum. Gent 18, 407–413. 10.1038/ejhg.2009.190 19888295 PMC2987250

[B21] Stabile M. Rispoli A. F. Capuozzo M. Ferbo U. Stabile G. (2023). Bifid cardiac apex and spongiform cardiomyopathy in fetus with small microdeletion 16p12.2 of paternal origin. Critical points in family communication on 16p12.2 microdeletion. Clin. Case Rep. 11, e7602. 10.1002/ccr3.7602 37405046 PMC10315447

[B22] Sugiyama K. Moteki H. Kitajiri S. I. Kitano T. Nishio S. Y. Yamaguchi T. (2019). Mid-frequency hearing loss is characteristic clinical feature of OTOA-associated hearing loss. Genes 10, 715. 10.3390/genes10090715 31527525 PMC6770988

[B23] Swetha J. Sakthignanavel A. Manoharan A. Rangarajalu J. Arunagiri P. Govindasamy C. (2025). A 250-kb microdeletion identified in chromosome 16 is associated with non-syndromic sensorineural hearing loss in a south Indian consanguineous family. J. Audiol. Otol. 29 (1), 31–37. 10.7874/jao.2024.00038 39916398 PMC11824526

[B24] Tassano H. PatriziaCalcagno A. F. Fiorio P. Gimelli G. Capra V. (2019). Distal 16p12.2 microdeletion' in a patient with autosomal recessive deafness-22. J. Genet. 98, 56. 10.1007/s12041-019-1107-0 31204719

[B25] Uppinkudru C. Basavaraju R. Udupi G. A. Mehta U. M. (2024). Schizophrenia in the context of neurodevelopmental disorders in 16p12.2 chromosomal deletion: a case report. Indian J. Psychol. Med. 46 (3), 283–284. 10.1177/02537176231222570 38699775 PMC11062308

[B26] Wang Y. Zhou H. Fu F. Liao C. Yu Q. Huang R. (2022). Prenatal diagnosis of chromosome 16p11.2 microdeletion. Genes (Basel) 13, 2315. 10.3390/genes13122315 36553582 PMC9778018

[B27] Watson C. T. Tomas M. B. Sharp A. J. Mefford H. C. (2014). The genetics of microdeletion and microduplication syndromes: an update. Annu. Rev. Genom Hum. G. 15, 215–244. 10.1146/annurev-genom-091212-153408 PMC4476258 24773319

[B28] Xiang J. Ding Y. Song X. Mao J. Wang T. Liu Y. (2020). Clinical utility of SNP array analysis in prenatal diagnosis: a cohort study of 5000 pregnancies. Front. Genet. 11, 571219. 10.3389/fgene.2020.571219 33240322 PMC7677511

